# Optimizing the sensitivity and resolution of hyaluronan analysis with solid-state nanopores

**DOI:** 10.1038/s41598-022-08533-1

**Published:** 2022-03-16

**Authors:** Felipe Rivas, Paul L. DeAngelis, Elaheh Rahbar, Adam R. Hall

**Affiliations:** 1 Virginia Tech-Wake Forest University School of Biomedical Engineering and Sciences, Wake Forest School of Medicine, Winston-Salem, NC 27101 USA; 2 Department of Biochemistry and Molecular Biology, University of Oklahoma Health Sciences Center, Oklahoma, OK 73104 USA; 3 Comprehensive Cancer Center, Wake Forest School of Medicine, Winston-Salem, NC 27157 USA

**Keywords:** Nanobiotechnology, Biosensors, Single-molecule biophysics

## Abstract

Hyaluronan (HA) is an essential carbohydrate in vertebrates that is a potentially robust bioindicator due to its critical roles in diverse physiological functions in health and disease. The intricate size-dependent function that exists for HA and its low abundance in most biological fluids have highlighted the need for sensitive technologies to provide accurate and quantitative assessments of polysaccharide molecular weight and concentration. We have demonstrated that solid state (SS-) nanopore technology can be exploited for this purpose, given its molecular sensitivity and analytical capacity, but there remains a need to further understand the impacts of experimental variables on the SS-nanopore signal for optimal interpretation of results. Here, we use model quasi-monodisperse HA polymers to determine the dependence of HA signal characteristics on a range of SS-nanopore measurement conditions, including applied voltage, pore diameter, and ionic buffer asymmetry. Our results identify important factors for improving the signal-to-noise ratio, resolution, and sensitivity of HA analysis with SS-nanopores.

## Introduction

Glycosaminoglycans (GAGs) are important macromolecules found ubiquitously in mammalian tissues and biofluids where they support diverse biomechanical and biochemical processes^1,2^. Hyaluronan (hyaluronic acid, or HA), is a linear anionic glycan, that has been identified as a promising biomarker for a variety of disease pathophysiologies and inflammatory processes. This importance stems from its involvement in functions ranging from innate immunity regulation^3^, to extracellular matrix structural support^1^, to lubrication and hydration of joints and tissues^2,4–6^. A critical aspect of HA is its size-dependent behavior^7^, wherein its biological function is strongly correlated to molecular weight (MW). For example, ‘high’ MW (greater than a few hundred kDa) HA has been shown to exert anti-inflammatory^2,7,8^, anti-angiogenic^2,7,8^, and anti-migratory^2,7–9^ behaviors, conversely ‘low’ MW (typically < 100 kDa) HA has been found to stimulate the expression of pro-inflammatory signals^2,7^, induce angiogenesis^2,7^, and promote cancer progression and invasion^10^. However, the exact transition(s) of the ‘high’ to ‘low’ MW size range as it pertains to biological function is still an active and sometimes contentious area of inquiry. Coupled with the implications of in vivo HA concentration over time for a variety of disease-specific conditions^6,11^, these attributes highlight the importance of analytical technologies to provide accurate and quantitative assessments of both HA MW and concentration, particularly from small mass/volume or limited biospecimens^1,2^.

Established biochemical approaches like enzyme-linked immunosorbent assays (ELISA) can provide sensitive quantitation of HA concentration, but cannot inform the size (i.e*.* MW) distributions of HA. Other methods such as size exclusion chromatography (SEC)^12^ and mass spectrometry^13,14^ can provide MW discrimination but are prone to practical challenges that include limited dynamic range^13,14^ and dependency on complex instrumentation with extensive sample pre-treatment before testing. Consequently, gel electrophoresis has been adopted as a standard approach for determining HA size^15–17^. However, while quantitatively robust^15–17^, a major limitation of gel electrophoresis is its large mass requirement; typically, ~ 2–4 μg of HA is needed per lane for visualization and even then MW subpopulations can be challenging to distinguish and compare.

**Figure 1:**
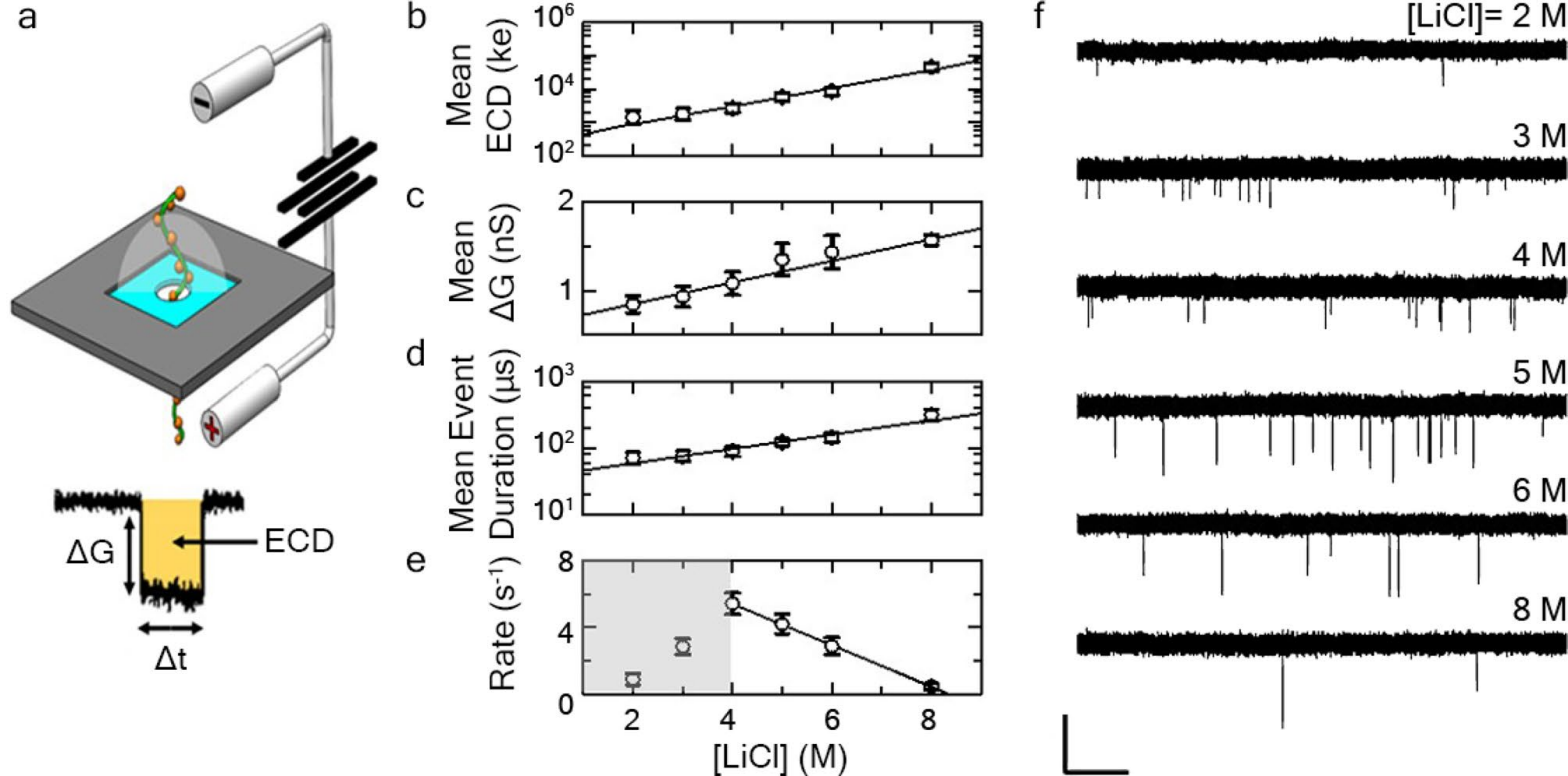
(**a**) Schematic representation of HA translocation through a SS-nanopore. Inset: typical event profile with amplitude (ΔG), event duration (Δt), and integrated area (ECD, shaded yellow) indicated. SS-nanopore results under varying symmetric LiCl concentrations, displaying mean values of (**b**) ECD, (**c**) conductance change, (**d**) event duration, and (**e**) event rate. Solid lines are exponential (**b**,**d**) or linear (**c**,**e**) fits to the data and error bars represent measurement standard deviation. All measurements were performed at an applied voltage of 200 mV using quasi-monodisperse 237 kDa HA at a concentration of 2.5 ng/µL using SS-nanopores with diameters of 7–9 nm. At least 1000 events were considered for each data point. (**f**) Representative current traces obtained at different symmetric LiCl concentrations (Top to bottom: 2, 3, 4, 5, 6, and 8 M). Scale bars represent 2 nS (vertical) and 250 ms (horizontal).

In response to these limitations, our laboratory recently proposed solid-state (SS-) nanopore technology as an alternative strategy for the quantitative analysis of HA^18^. The SS-nanopore platform consists of a nanometer-scale opening in a thin-film membrane that divides two solvent compartments filled with electrolyte solution (Fig. 1a). The application of a voltage across the membrane sets up an electric field that allows the steady passage of ions through the pore and generates a stable current. As individual HA molecules are drawn electrophoretically through the same opening, the polymers produce temporary resistive pulses (or “events”) in the signal as the ions are blocked (Fig. 1a, inset), the properties of which can provide direct biophysical information about the transiting molecules. This approach is motivated by past work employing biological nanopores for this purpose^19^ and is closely related to other recent reports of SS-nanopores being utilized to probe GAGs like chondroitin sulfate and heparin^20,21^.

Various event characteristics (amplitude, duration, and others) have been employed to study aspects of DNA^22^, RNA^23^, and proteins^24–26^, but the two factors that have been shown to be the most informative for HA analysis in particular are integrated event amplitude (Fig. 1a, inset; also known as event charge deficit^27^, or ECD) and event frequency, which correspond to the MW and concentration of the analyte, respectively^18^. However, the accuracy of determining these values depends critically on understanding the impacts of experimental parameters. For example, the temporal resolution of the instrument^28,29^ creates an intrinsic limitation for detecting smaller HA chains because of their brief residence time in the sensing region of the nanopore. Likewise, probing low analyte concentrations can necessitate long measurement times and/or create the need for a low noise floor that may be difficult to achieve. A range of modifications to conventional SS-nanopore detection have been demonstrated to improve signal-to-noise ratio (SNR), reduce translocation speed, or increase capture rate. These factors have included optimization of nanopore dimensions^23,30^ and material^31,32^ as well as alteration of buffer conditions^33–35^. However, any exploration of such experimental variations has so far been performed for nucleic acids and proteins but not GAGs.

In this work, we present a systematic investigation of the effects of experimental conditions on HA analysis with SS-nanopores. Using a series of synthetic HA polymers each having a known, narrow size distribution (quasi-monodisperse), we investigate the effects of salt concentration, applied voltage, and nanopore diameter on critical translocation event properties. We then employ salt gradients across the SS-nanopore membrane to explore the impact of asymmetric ionic conditions on measurement sensitivity. Our results map out the consequences of varying measurement conditions, ultimately improving our understanding of the SS-nanopore measurement approach and enhancing its efficacy for quantitative and sensitive HA analysis.

## Results and discussion

The ability to improve sensitivity and optimize MW resolution is essential for HA analysis with SS-nanopores. Past studies^35^ on DNA have shown that modifying solvent ionic strength can alter capture rates and improve SNR. Salt concentration has also been varied to differentiate GAGs from synthetic mixtures^36^. Consequently, we first focused on electrolyte concentration as a solvent condition that can be readily adjusted in our existing system. As in our previous work^18^, we employed LiCl as the electrolyte because of its extraordinary solubility in water and the small size of the Li^+^ cation that enables efficient screening of charged molecules^35^. These characteristics reduce the net charge of translocating molecules, lowering the driving force at a given applied voltage and reducing the translocation velocity (i.e. increased dwell time), both of which are favorable for resolution.

To probe the effects of salt concentration, we conducted a series of translocation experiments using SS-nanopores with diameters ranging from 7 to 9 nm. All measurements were performed using quasi-monodisperse HA with a mean MW of 237 kDa (~ 1190 monosaccharide units; concentration of 2.5 ng/μL) and 200 mV applied voltage for consistency with previous work^18^. We varied the LiCl concentration of the measurement buffer from 2–8 M (below 2 M, low SNR prevented efficient detection of translocations) and observed changes in several event properties (Supplementary Fig. S1). First, mean ECD increased exponentially with salt concentration (Fig. 1b). Separating this metric into its constituents, we found that event amplitude varied linearly with LiCl content, changing ~ 3-fold from 2 to 8 M (Fig. 1c), while event duration depended exponentially on concentration, increasing by nearly an order of magnitude over the same range (Fig. 1d). These findings were concomitant with the effects of salt concentration established from previous reports on DNA^35^: higher salt concentrations provide more ions to contribute to the measured ionic current and a proportionally greater blockage by the translocation of a molecule and also provide better screening of the negatively charged analytes and SS-nanopore walls, altering the electrophoretic driving force and thus the event duration.

While the increase in event duration for HA is in qualitative agreement with the measurements of Kowalczyk et al. on DNA, that earlier report found a linear dependence on LiCl concentration^35^, contrasting the exponential response observed here. This may suggest additional factors at play, potentially including electroosmotic effects as well as the reduced radius of gyration of the HA that is driven both by its low persistence length^37,38^ (about an order of magnitude smaller than that of double-strand DNA^39^) and the high molecular compaction of the polymer chain under elevated charge screening due to low self-avoidance (HA charge density is approximately 1/6 that of double-stranded DNA^40^). These elements could result in a different translocation regime altogether and future studies may be able to elucidate this further. However, we also note that our measurements were performed over a different range of LiCl (i.e. 2–8 M) than was used for DNA (i.e. 0.5–4 M), potentially revealing more details of the translocation dynamics than were observed in the past report.

The improvements in resolution observed with increasing LiCl concentration suggested that HA analysis should be performed under the highest ionic strength achievable. Yet, in addition to these changes in event characteristics, we also measured a strong reduction in event rate at higher electrolyte concentration (Fig. 1e,f). This observation could be explained by the same charge screening described above: a reduction of the effective HA charge would be expected to impact electrophoretic driving force and thus lower capture efficiency at a given bias. We note that the lower event rates measured for 2 and 3 M LiCl was likely attributable to reduced SNR causing some events to be missed entirely. Indeed, no significant events were observed for 237 kDa HA when using LiCl at concentrations lower than 2 M (or with 1 M concentrations of either KCl or NaCl for the same reason). Because the need for improving MW resolution (i.e. high SNR) must be balanced with the need for sensitivity (i.e. detection of low concentration of analyte), we elected to employ the moderately high salt concentration of 6 M LiCl for further measurements unless otherwise noted, as it still provided a balance of reasonable event rate with overall favorable translocation event properties.

**Figure 2:**
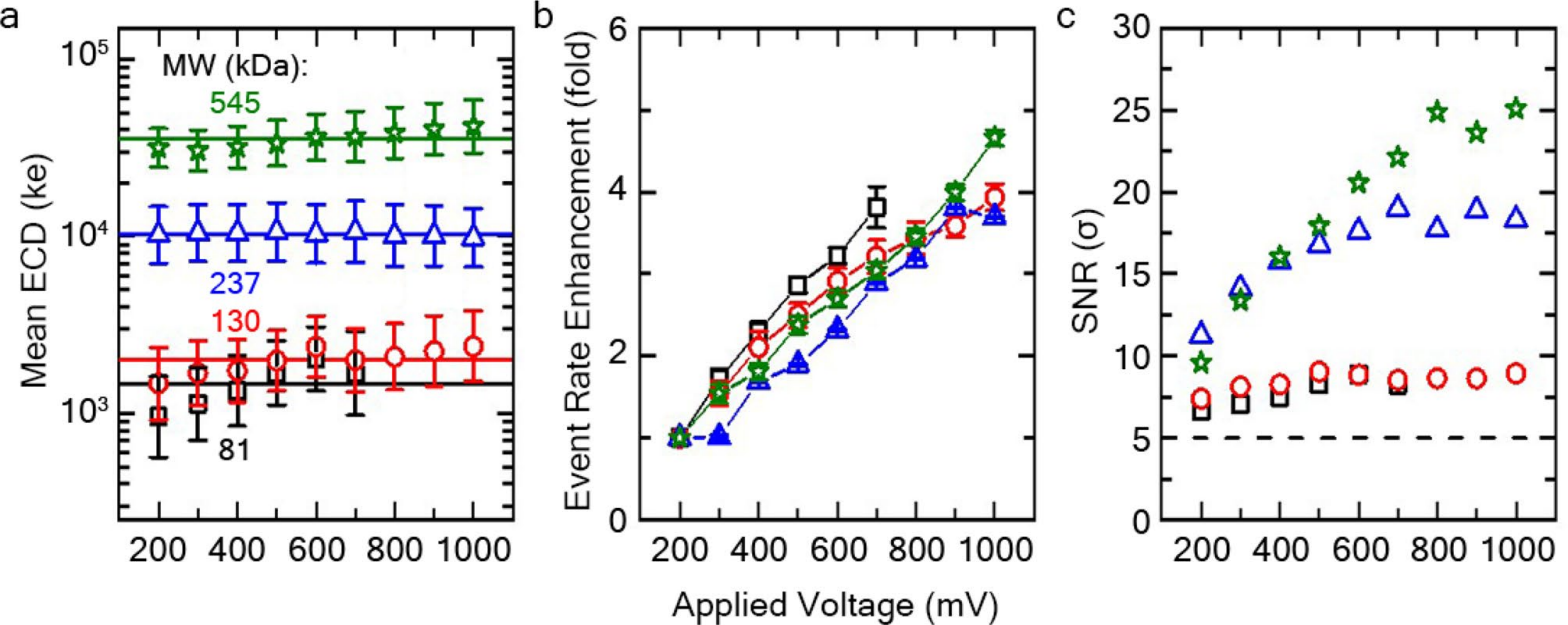
(**a**) Mean ECD of individual quasi-monodisperse HAs as functions of applied voltage. Lines represent the average value for each MW across all voltages. (**b**) Voltage-dependent event rate enhancement (relative to rate at 200 mV) for all MWs. (**c**) SNR for each MW across all voltages. Dashed line is threshold used for analysis. Colors: 81 (black squares), 130 (red circles), 237 (blue triangles), 545 kDa (green stars). Results collected using a single SS-nanopore (7.5 nm diameter) representing at least 2000 events for each data point. HA concentrations between 2.5–5 ng/μL were used. Error bars represent measurement standard deviation.

Since the voltage applied across a SS-nanopore has a fundamental impact on translocation characteristics, we next sought to investigate these changes by performing a series of measurements under varying biases. Initial assessments were performed using quasi-monodisperse HAs with mean MW of 81, 130, 237 and 545 kDa, respectively, across a broad voltage range of 200–1000 mV. All measurements for this data set were collected using a single 7.5 nm diameter pore for consistency. In previous work^18^, we showed that quasi-monodisperse HA polymers produced events with a narrow population of ECDs corresponding to their mean MW, producing distributions similar in form to an electropherogram. Our voltage-dependent analyses here confirmed this distinction but further showed a remarkably consistent mean ECD value for each MW across the voltage range tested (Fig. 2a). Note that while this particular nanopore device failed before completing the full voltage range for 81 kDa (Fig. 2a, black), the data obtained up to 700 mV showed similar consistency as other MWs. This invariance suggested that any increase in event amplitude (i.e. SNR) at higher voltages was compensated by the reduced translocation time (Supplementary Fig. S2). However, the results did not immediately suggest a clear advantage for any particular voltage condition. For example, the separation between the two lowest MWs studied here (81 and 130 kDa) was equally narrow under all conditions.

Figure 2b shows the enhancement of mean event rates (relative to 200 mV) when HA concentration was held constant for each independent MW. From these data, we found an increase in average molecular capture rate for higher applied voltages, similar to past reports using other molecular targets^41,42^. As with our previous work^18^, the consistent linear relationships indicated a diffusion-limited translocation regime^34^ and highlighted the absence of a MW preference for HA capture^43^; a critical observation that substantiated the capacity of the SS-nanopore platform to deliver an accurate size distribution for polydisperse mixtures. We note that neither of the above findings were strongly impacted by operating bandwidth (Supplementary Figs. S3 and S4) except that reduced total event rates were observed for small MW HAs, reflecting the loss of signal that accompanied an increased low-pass filter frequency (*f*_*c*_).

Consequently, while the invariance of mean ECD did not identify a preferable voltage for HA analysis, the observed relative capture rate enhancement suggested improved sensitivity (i.e. the ability to evaluate a smaller net amount of HA) at higher bias. In addition, measured SNR was also found to improve with voltage (Fig. 2c) for all samples but particularly for HMW HA, further supporting a benefit to high-voltage measurements. However, these advantages must be balanced against the potential drawback of increased device fouling and clogging, which were observed to be more frequent at higher voltages. While typically reversible, such occurrences may limit nanopore lifetime and ultimately reduce measurement efficacy and throughput. To avoid this complication, we chose to employ low voltage conditions (200 mV) for all further measurements here.

Like voltage, SS-nanopore diameter is known to have a strong influence on the translocation event characteristics. For example, extremely small (< 5 nm) nanopores have been studied as a means for high-precision nucleic acid detection^44^, including showing efficacy for differentiating homopolymers^45^. To this end, we sought to study the effects of varying pore diameter on HA detection. A series of translocation measurements using SS-nanopores ranging in size from 4.5 to 19.0 nm were performed. Diameters were determined using the open pore conductance, *G*_*o*_, derived from an current–voltage (I–V) curve of each pore and the expression^23^.

1 $$G_{o} = \sigma \left[ {\frac{4L}{{\pi d_{p}^{2} }} + \frac{1}{{d_{p} }}} \right]^{ - 1} ,$$

where σ is bulk conductivity of the solution, *L* is the effective length of the pore, and *d*_*p*_ is the diameter. For the 6 M LiCl conditions employed, 19.4 S m^−1^ was used^46^ for σ. *L* was taken as 6.7 nm, following the convention^23^ of using 1/3 of the full membrane thickness (20 nm).

**Figure 3:**
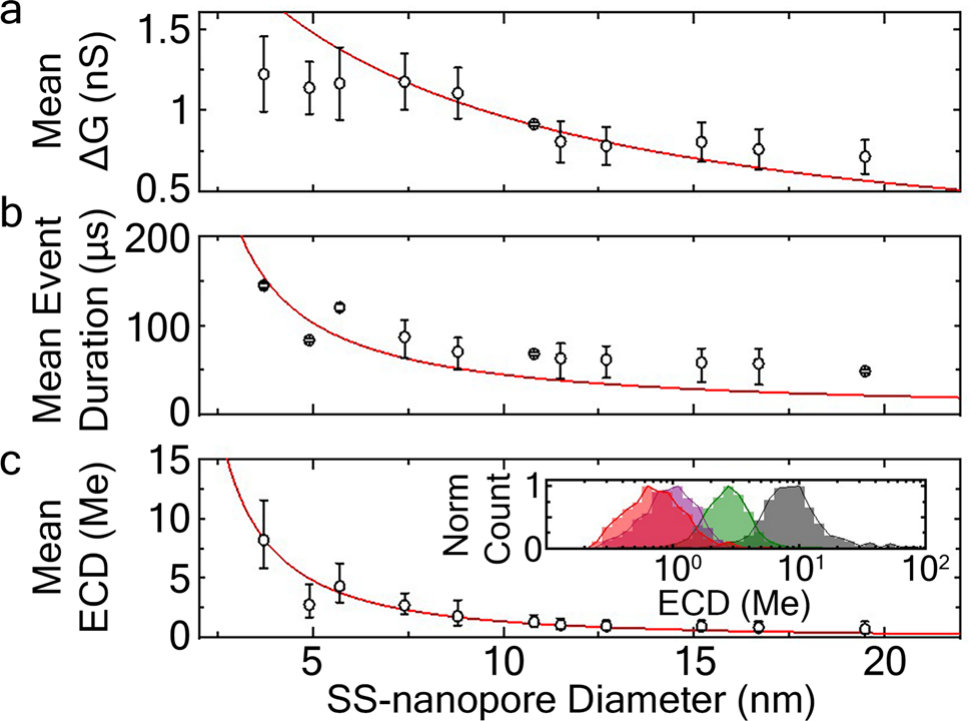
Effect of SS-nanopore diameter on mean event (**a**) conductance change, (**b**) duration, and (**c**) ECD values for 130 kDa quasi-monodisperse HA, at a concentration of 15 ng/μL. At least 1500 events were considered for each data point shown. Red lines are model fits to the data (see text for details). Error bars represent measurement standard deviation. Inset of (**c**) shows example normalized ECD histograms obtained from SS-nanopores with diameters (left to right) 19.5 (red), 12.9 (purple), 7.4 (green), and 3.7 nm (grey).

We initially probed 130 kDa quasi-monodisperse HA (15 ng/µL). From these measurements (Supplementary Fig. S5), we first found that the amplitude *ΔG* of translocation events decreased with pore diameter (Fig. 3a), in qualitative agreement with past experiments using both colloids^47^ and DNA^48^. To describe this reduction, a well-established series resistance model^47–51^ could be applied by calling *ΔG* the difference between the open pore conductance, *G*_*o*_, and the conductance of the partially blocked pore, *G*_*b*_. Because the diameter of the blocked pore is defined as (*d*_*p*_^2^* − d*_*HA*_^2^)^1/2^, where *d*_*HA*_ is the hydrodynamic diameter of the HA molecule, the full expression relating *ΔG* to nanopore diameter could be written as

2 $${\Delta }G = \sigma \left( {\left[ {\frac{4L}{{\pi d_{p}^{2} }} + \frac{1}{{d_{p} }}} \right]^{ - 1} - \left[ {\frac{4L}{{\pi \left( {d_{p}^{2} - d_{HA}^{2} } \right)}} + \frac{1}{{\sqrt {d_{p}^{2} - d_{HA}^{2} } }}} \right]^{ - 1} } \right).$$

Fitting this expression with *d*_*HA*_ as the only free parameter resulted in a curve that reasonably captured the trend of our data (Fig. 3a, red line). While differences may be accounted for by the non-cylindrical shapes of the pores^48^, a slight difference in their effective length *L* compared to the assumed value, or the folded conformations of translocating molecules themselves, the fit yielded a value for *d*_*HA*_ of 1.12 ± 0.01 nm, very close to the average diameter measured from the NMR structure of HA^52,53^ (0.8 nm).

Similarly, we found that event durations for the 130 kDa HA also decreased with diameter (Fig. 3b). This result was somewhat counterintuitive if only electrical forces were considered; since driving force (i.e. the sum of electrophoretic and electroosmotic forces) is known to reduce with pore diameter^54^, one might expect an increase in transit time with pore size. However, the downward trend could be understood by considering frictional drag between the HA and the SS-nanopore^55^, described by *A*(*d*_*HA*_/(*d*_*p*_* − d*_*HA*_)) where *A* is a scaling factor that captures the friction coefficient of the interaction and the velocity of the translocating molecule. Setting *d*_*HA*_ from above (1.12 nm) and varying *A* as a free parameter, we arrive at a fit that describes the shape of the raw data well (Fig. 3b, red line). The slight divergence at higher diameters may be due to the first order approximation that average translocation speed does not vary with *d*_*p*_ since there is experimental evidence of non-ideal translocations as pore size increases^56^.

We finally turned to ECD, which as a metric is the product of event amplitude and duration. Predictably from the trends of each constituent factor, the mean ECD value was also found to shift towards lower values as SS-nanopore diameter was increased (Fig. 3c). Combining the expressions used above and employing the same values for their respective fit parameters, we find excellent agreement with the analytical data (Fig. 3c, red line). Practically, this finding did not suggest a significant benefit to any particular SS-nanopore diameter, but instead simply highlighted the importance of taking pore diameter into account. However, coupling the observed ECD trend with changes in the electrical noise of the baseline signal demonstrated that increasing pore diameter was also accompanied by concomitant decrease in measurement SNR (Supplementary Fig. S6a), suggesting that smaller diameter SS-nanopores may be preferable.

**Figure 4:**
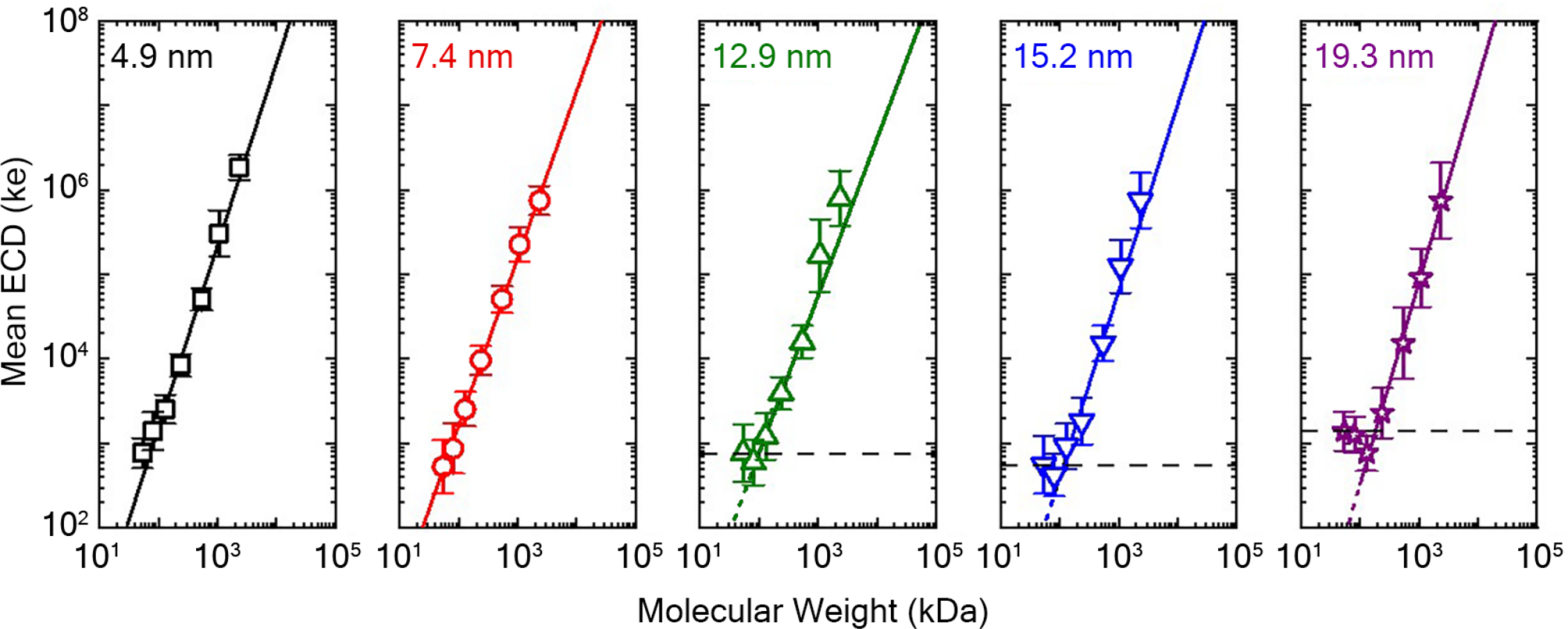
Mean ECD vs. quasi-monodisperse MW for SS-nanopores of various diameter [left to right: 4.9 (black squares), 7.4 (red circles), 12.9 (green triangles), 15.2 (blue inverted triangles), 19.3 nm (purple stars)]. Solid lines are power law fits (of the form y = b + x^α^) to the data down to the apparent noise floor (dashed line). A comparison of the fit exponent α across conditions is shown in Supplementary Fig. S7b (inset).

A critical aspect of HA analysis with SS-nanopores is the determination of the MW distribution via a calibration curve relating mean ECD to MW for different quasi-monodisperse HA specimens^18^. Therefore, it is important to consider the effect of nanopore diameter across a range of MWs rather than a single HA size only. Consequently, we expanded our next measurements to assess the impact of nanopore diameter on the established^18^ power law relationship between HA MW and ECD. For this assessment, we obtained ECD distributions from quasi-monodisperse HAs ranging in size from 54 kDa to 2.5 MDa using SS-nanopores with diameters of 4–20 nm (Supplementary Fig. S7a) and plotted their mean values with errors denoting standard deviation (Fig. 4).

Several observations arose from these data. First, the same power law relationship was maintained across all pore diameters (Supplementary Fig. S7b), yielding an average exponent *α* of 2.02 ± 0.16 (Supplementary Fig. S7b, inset). Indeed, plotting the ECD against nanopore diameter for discrete HA MWs produced nearly identical dependencies (Supplementary Fig. S8). As a result, calibrations can be inferred for all intermediate diameters, enabling better precision in MW determination. Second, ECD error (i.e. the standard deviation of ECD distribution) for a given MW increased with nanopore diameter. We note that distribution widths were influenced by both the electrical noise of the measurement and the actual polydispersity of the HA (see “Materials and methods”), the latter of which was constant for each MW. Consequently, the increasing standard deviations with pore size were indicative of a negative impact on intrinsic measurement accuracy. Third, the MW resolution was adversely affected by an increase in nanopore diameter. A distinct departure from the power law trend was observed at lower MW HA, most easily seen by contrasting the data obtained with a 19.3 nm diameter pore—where MW ≤ 130 kDa yielded unchanging ECD values (Fig. 4, right)—with measurements on a 4.9 nm pore, which showed a consistent trend down to 54 kDa (the smallest MW studied here, Fig. 4, left). Considering all of these factors, these data again suggested an advantage for smaller diameter SS-nanopores. However, it is important to acknowledge that small nanopores also tended to result in more observable device fouling and clogging, which were nearly absent for larger nanopore diameters. This trade-off indicated that utilizing SS-nanopores of intermediate diameter (~ 7 nm) would be ideal in practice, as was done in our previous research^18^.

**Figure 5:**
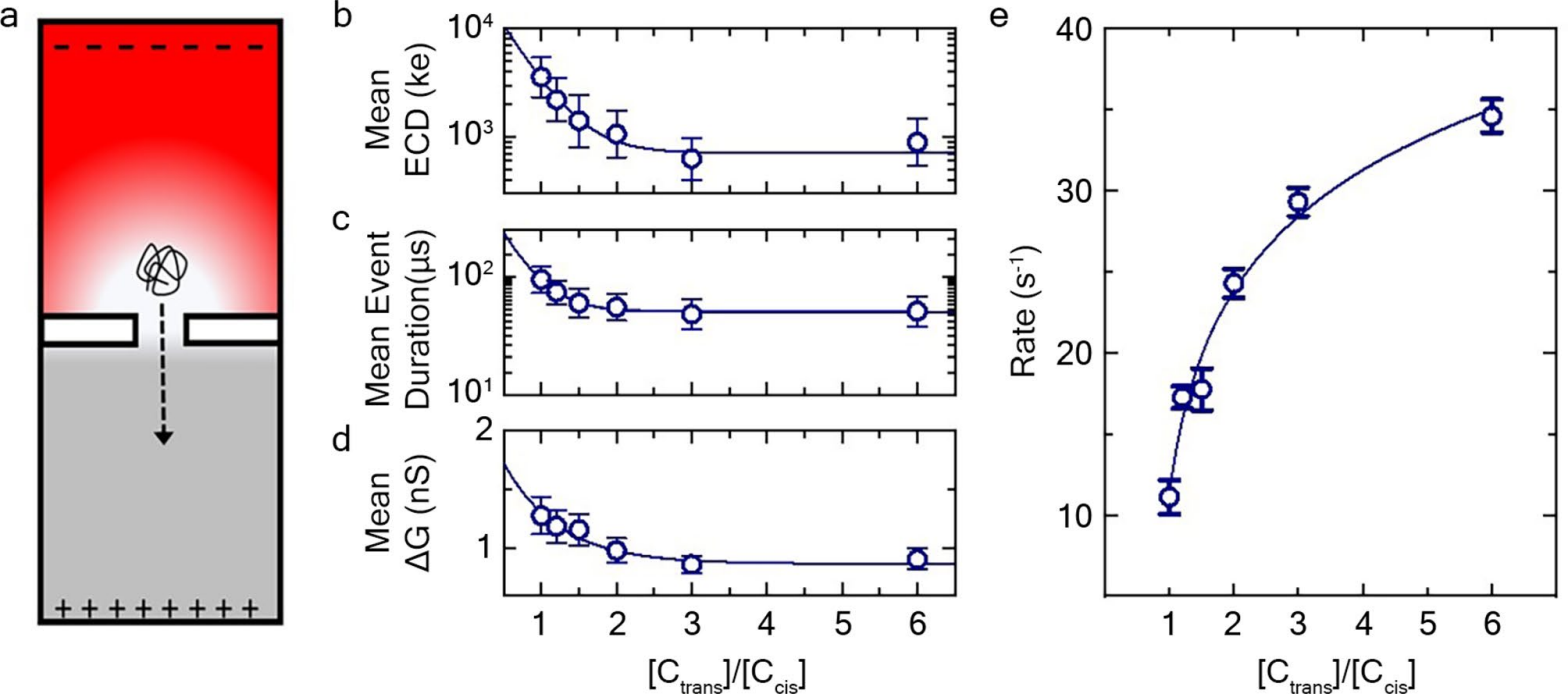
(**a**) Schematic representation of electric field across a SS-nanopore under asymmetric salt conditions (dash arrow indicates movement of HA molecule from *cis*- to *trans*-chamber). Translocation results under varying LiCl asymmetry where C_*trans*_ is kept at 6 M and C_*cis*_ is varied from 1 to 6 M, showing mean ECD (**b**), event duration (**c**), and conductance change (**d**). Solid lines are exponential fits to the data. (**e**) Event rate across asymmetric buffer conditions [C_*trans*_:C_*cis*_] LiCl. Solid line is a logarithmic fit to the data. All measurements were taken at an applied voltage of 200 mV using quasi-monodisperse 237 kDa HA samples prepared at a 2.5 ng/µL concentration. Results collected using SS-nanopores 7–9 nm in diameter representing at least 1000 events for each data point. Error bars represent measurement standard deviation.

Having established the impacts of device dimensions on HA detection, we finally revisit the effects of solvent conditions by investigating salt asymmetry. It has been shown previously that the use of a salt gradient—specifically a higher salt concentration on the *trans-*side of the SS-nanopore membrane than on the *cis-*side (Fig. 5a)—can have significant impacts on translocation dynamics^34^, including increasing the capture rate^57^. This effect has been attributed to imbalanced ion pumping under bias that results in the accumulation of positive charges around the pore entrance on the *cis*-side, effectively extending the electric field farther into solution and promoting higher capture efficiency^34^. Because enhancing the event rate will enable greater sensitivity (i.e. robust analysis from a smaller total mass of HA), we performed a set of measurements under asymmetric transmembrane salt conditions.

SS-nanopore translocations were carried out using 237 kDa quasi-monodisperse HA while maintaining *trans*-side buffer conditions at 6 M LiCl and varying *cis*-side LiCl concentration from 1 to 6 M. From these experiments, we observed an exponential decrease in ECD as the *trans*:*cis* ratio was increased (i.e. as *cis*-concentration was reduced; Fig. 5b). Considering the constituents of these data, we found that the observed effect was dominated by an exponential decrease in dwell time (Fig. 5c; note the semi-log axes) while event conductance change reduced only modestly (Fig. 5d). This result was in sharp contrast to a previous SS-nanopore study^34^ using DNA that reported event durations in fact increase with an approximately linear trend as *trans*:*cis* asymmetry is increased.

It has been explained^34^ that a higher *trans-*chamber salt concentration will create cation selectivity under the influence of a driving potential. As the positively charged ions are driven through the nanopore, they build up at the *cis-*opening and induce polarization. This perturbation of the electric field creates an asymmetric extension of the voltage drop far into the solution on the *cis-*side of the membrane, effectively reducing the potential profile through the pore itself and thus yielding slower translocation durations. A key factor in our work was the use of LiCl as the electrolyte. The small size of the Li^+^ cation promotes efficient screening of charged molecules that is enhanced by its extraordinary solubility in water, enabling high concentrations to be utilized. As described above in our investigation of symmetric salt conditions (and elsewhere^35^), this reduces the net charge of translocating molecules, resulting in a lower driving force and lower translocation velocity (i.e. increased event duration). Consequently, there were two competing effects as *trans*-side LiCl concentration was maintained at 6 M and *cis*-side concentration was reduced: (1) potential profile manipulation caused by increasing asymmetry, serving to curtail the voltage drop across the pore and thus increase event durations; and (2) reduced charge screening driven by the decreasing (effective) ionic strength at the pore, serving to enhance driving force and decrease event duration. Critically, the former scales linearly with *cis-*concentration^34^ while the latter varies exponentially (c.f*.* Fig. 1d). As a result, our data showing that event duration decreased strongly with asymmetry suggested that the charge screening effect was dominant in our compared than the potential profile alteration.

To test this premise, we performed additional measurements wherein the *cis-*side LiCl concentration was kept at 4 M to maintain constant charge screening while the *trans-*side concentration was increased from 4 to 8 M to achieve salt asymmetry. Under these conditions, we instead observed a linear increase in mean ECD (Supplementary Fig. S9a, top panel); an effect that was comprised of similar increases in both dwell time and amplitude (Supplementary Fig. S9a, middle and lower panels). Consequently, when differences in screening on the *cis-*side were negated, our results were in agreement with expectations from the cationic selectivity effect alone and thus past reports^34^.

Notably, we observed a significant rate enhancement effect for all data relative to *C*_*trans*_:*C*_*cis*_ ratio, well-described by logarithmic trends (Fig. 5e and Supplementary Fig. S9b). While Wanunu et al*.* reported^34^ a linear relationship between rate enhancement and salt asymmetry, it was only observed for ratios above approximately *C*_*trans*_:*C*_*cis*_ = 1.5. It is likely that a similar trend would emerge from our measurements, but we were more limited in the range of attainable asymmetry because *cis-*side LiCl concentrations < 1 M yielded no detectable events under our conditions and *trans-*side LiCl concentrations > 8 M resulted in current instabilities that made measurements challenging. Moreover, our results indicated that the benefit in enhanced event rate achieved through asymmetric salt concentrations should be balanced against the cost in resolution resulting from reduced ECD relative to high molarity symmetric conditions. Event properties for all asymmetric measurement conditions are shown in Supplementary Fig. S10.

## Conclusions

HA is an emerging biomarker for diverse disease pathologies that features critical size-dependent biological functions and in vivo activity that is tied to its concentration^7^. Therefore, it is critical to demonstrate high-quality, reliable, and reproducible analysis of HA size distribution and abundance. Towards this goal, we have presented a thorough study of how diverse experimental conditions affect the sensitivity and resolution of HA analysis with SS-nanopores using synthetic quasi-monodisperse HA. First, we investigated the impact of ionic strength. We observed that increasing LiCl concentration of the measurement buffer resulted in an improvement in ECD signal (i.e*.* resolution) that was accompanied by a reduction in event rate (i.e. sensitivity). This trade off suggested that intermediate concentrations (~ 6 M) were optimal for HA analysis. Varying applied bias, we found that ECD values did not change significantly across a broad range of 200–1000 mV even while event rates increased predictably with voltage. However, this was accompanied by a decrease in measurement precision for both values, meaning that the relative importance of sensitivity and accuracy to a measurement should be considered. We next considered how SS-nanopore diameter affected translocation signal characteristics, determining that measurement resolution was improved with small pores. When balanced with the increased observable prevalence of fouling at low diameter, we concluded that moderately small (~ 7 nm) pores should be targeted. Additionally, the predictable variation of the calibration relationship between ECD and MW across conditions also circumvented issues related to natural pore-to-pore variability. Finally, we explored the impacts of asymmetric transmembrane salt conditions. Through these data, we demonstrated that capture rates could be improved significantly (i.e. increased sensitivity), but at the cost of ECD and thus resolution.

Collectively, our results can be used to tailor measurement conditions for a given application. For example, when probing synthetic HA or HA derived from abundant specimens where sample mass is not limited, low-voltage measurements with small diameter pores and symmetric, high molar salt concentrations are preferable to maximize resolution. In contrast, for specimens where HA is sparse^7^, increased voltage or salt asymmetry would allow for better minimum mass sensitivity—already 10 ng from our previous work^18^—though at the expense of some resolution. Expanding buffer conditions also provides a potential route to additional applications, such as studies that require low salt conditions while retaining measurement efficacy. Considering the challenges of conventional analytical measurements of HA pertaining to analyte size limitations, sample mass requirements, and ease of use, our systematic investigation of experimental parameters impacting SS-nanopore detection demonstrates the potential of our platform as a glycan assessment technology.

## Materials and methods

### HA samples

A total of seven discrete, quasi-monodisperse HA samples were used (Hyalose, LLC, Oklahoma City, OK) having average MWs of 54, 81, 130, 237, 545, 1000, or 2500 kDa, respectively^58^. The MW distributions of the synthetic HA ranged < 5% from their reported means (polydispersity = 1.001–1.035) as determined by multiangle laser light scattering size exclusion chromatography (MALLS-SEC). Lyophilized samples were resuspended in 1X phosphate buffered saline (137 mM NaCl, 2.7 mM KCl, 8 mM Na_2_HPO_4_, and 2 mM KH_2_PO_4_, pH ~ 7) to a concentration of 1 μg/μL (approximated from the measured mass of lyophilized HA) to create stock solutions and diluted as needed for measurements. All samples were stored at 4 °C prior to use.

### SS-nanopore measurements

Each solid-state nanopore device consisted of a single aperture in a 19 nm thick silicon nitride thin-film membrane supported by a 4-mm silicon chip (Norcada, Inc. Alberta, Canada). Nanopore fabrication was performed by He ion milling (Orion PLUS, Carl Zeiss, Peabody, MA) using a method described elsewhere^59^ through which calibrated ion doses were used to produce pores of different target diameters with a precision of ± 2–3 nm. This approach offers higher precision and resolution than Ga focused ion beam milling^60^, better throughput than traditional transmission electron beam fabrication^61^, and better flexibility (e.g*.* array formation and shape/location control) than controlled breakdown techniques^62^. Chips were stored in 50% ethanol prior to measurement, at which time they were rinsed with ethanol and water, dried under filtered air flow, and treated with air plasma (30 W, Harrick Plasma, Ithaca, NY) for 2 min on each side before being loaded into a custom flow cell produced by 3D printing (Carbon, Redwood City, CA). After immediate introduction of an initial measurement buffer (6 M LiCl, 10 mM Tris, 1 mM EDTA, pH 8.0), Ag/AgCl electrodes (Sigma-Aldrich, St. Louis, MO) were positioned in each chamber for voltage application and ionic current measurement using a patch-clamp amplifier (Axopatch 200B, Molecular Devices, Sunnyvale, CA). Measured current was used to confirm low-noise baseline signal as well as a linear I-V curve from which SS-nanopore diameter was derived using an established model described in Eq. (1) in the text.

For all measurements, buffers consisted of 10 mM Tris, 1 mM EDTA, pH 8.0 with a LiCl molarity as indicated in the text. Using a target measurement buffer, quasi-monodisperse HA was diluted from the stock solution to the working concentration indicated in the text and then introduced to the *cis*-chamber in an approximate volume of 12 µL. HA concentrations were chosen to minimize total measurement time (i.e. adequate event rate) and were held constant when event rates were compared between conditions. A bias was then applied across the membrane to record trans-pore ionic current at a rate of 200 kHz using a 100 kHz four-pole Bessel filter. An additional 5 kHz low-pass filter was applied using custom software unless otherwise noted. Events caused by translocations of HA through the pore into the opposite (*trans-*) chamber were identified as transient reductions in the ionic current > 5σ in amplitude compared to baseline noise and with durations in the range of 25–2.5 ms. Data were recorded in discrete blocks (3.2 s duration) from which the mean and standard deviation of event rates were determined by direct counting^63^.

### Varying salt conditions

When varying ionic strength in one or both chambers for a single SS-nanopore device, measurements were conducted from the lowest ionic concentration to the highest to avoid effects of residual salt, flushing with several volumes to replace the fluid completely between measurements. For asymmetric salt measurements, the voltage offset of the amplifier was adjusted for each new condition to zero the measured current for an applied voltage of 0 mV. ECD values were determined for each molecular translocation event by calculating the integrated area defined by its current signature (see Fig. 1a). Mean ECD values were determined from log-normal (Gaussian on a log scale) fits to the ECD distribution for an individual pore measurement (c.f. Fig. 3c, inset). For large MW quasi-monodisperse HA samples (≥ 1000 kDa), background signal was observed that was attributed to sample fragmentation (see Supplementary Fig. S7a), likely induced by sample handling or storage. For these, a multi-peak fit analysis was performed, and the most prominent peak was used.

## Supplementary Information

Supplementary Figures.
