# Supplementary material for: Optimizing the sensitivity and resolution of hyaluronan analysis with solid-state nanopores
Source: Sci Rep. 2022 Mar 16;12:4469. doi: 10.1038/s41598-022-08533-1 (PMC8927330; doi:10.1038/s41598-022-08533-1)
Supplement: Supplementary file 1 — Supplementary Figures. [file 41598_2022_8533_MOESM1_ESM.pdf]

***Supplementary Information:***
**Optimizing the Sensitivity and Resolution of Hyaluronan Analysis with Solid-State Nanopores**

*Felipe Rivas, Paul L. DeAngelis, Elaheh Rahbar, and Adam R. Hall*

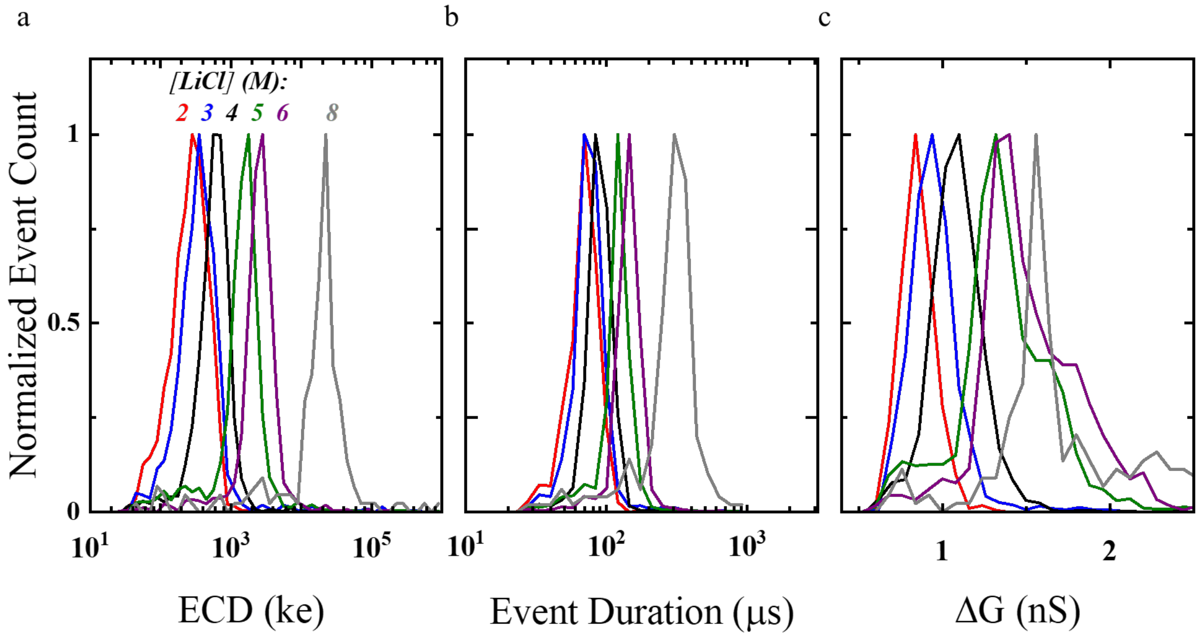


***Supplementary Figure S1.*** *Representative distributions of ECD (a), event duration (b), and mean event amplitude (d) for 237 kDa quasi-monodisperse HA while varying the symmetric LiCl salt concentration (Left to right: 2, 3, 4, 5, 6, and 8 M). All measurements were obtained using SS-nanopores of 7-9 nm in diameter under an applied voltage of 200 mV at a sample concentration of 2.5 ng/µL concentration. Results collected using SS-nanopores of 7-9 nm in diameter and at least 2,000 events were considered for each data point.*

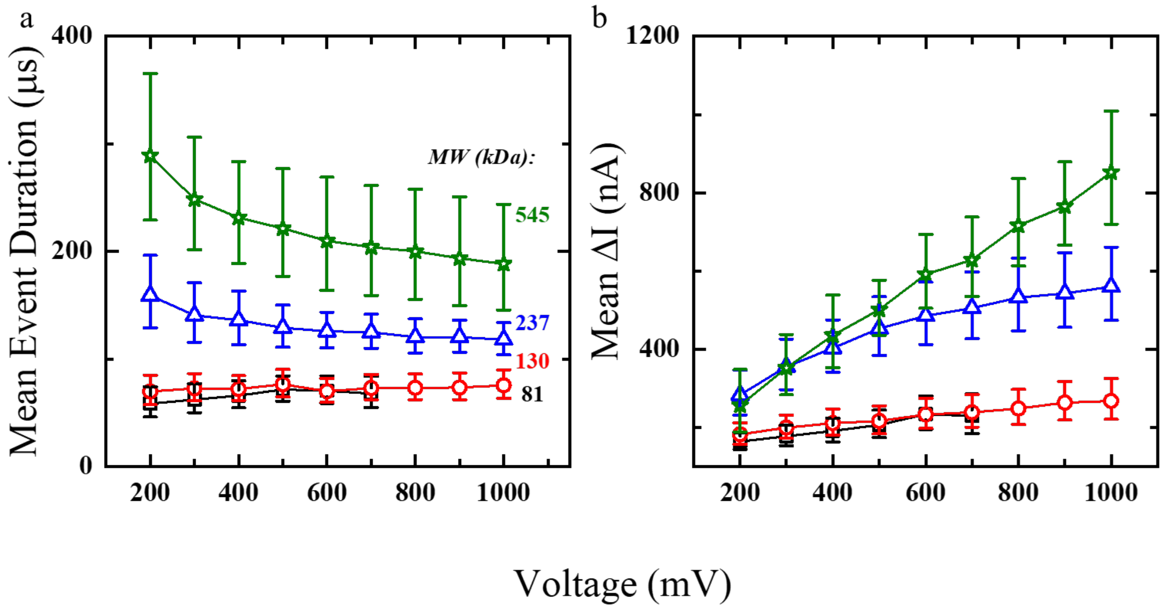


***Supplementary Figure S2.*** *Mean Event duration (a) and signal amplitude (b) values obtained from the log-normal distributions of individual quasi-monodisperse HA samples measured at different voltages using a low-pass filter frequency (fc) of 5 kHz. Colors represent 81 (black squares), 130 (red circles), 237 (blue triangles), or 545 kDa (green stars) HA. Results collected using a single SS-nanopore of 7.5 nm in diameter and at least 2,000 events were considered for each data point. Concentration of HA varied between 2.5-5 ng/µL for the different MW. Error bars represent measurement standard deviation.*

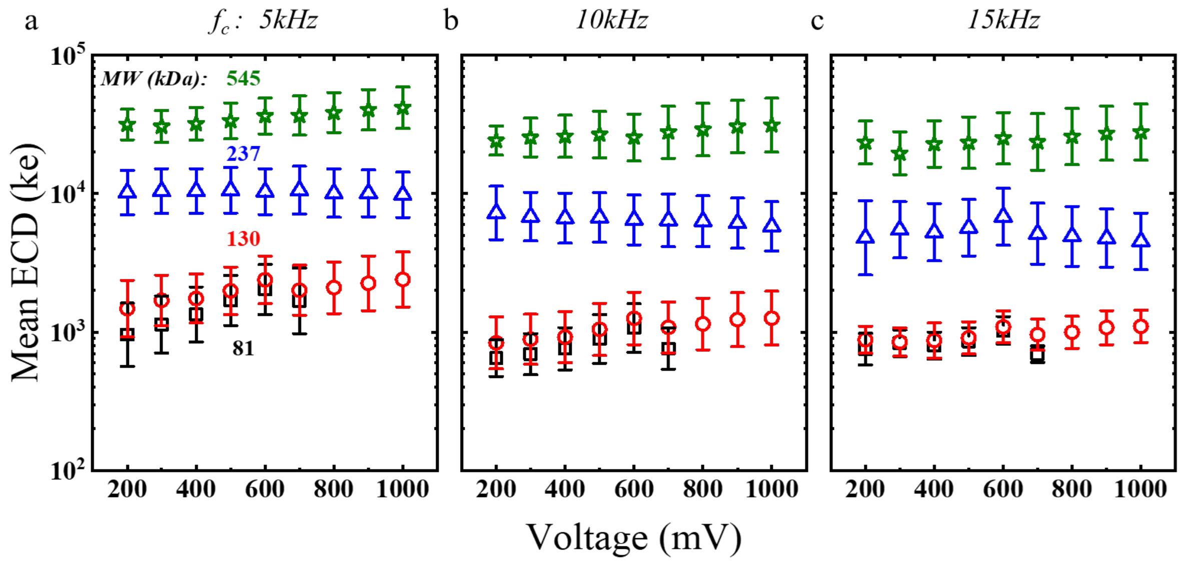


***Supplementary Figure S3.*** *Mean ECD values obtained from the log-normal distributions of individual quasi-monodisperse HA samples measured at different voltages using low-pass filter frequencies ($f_c$) of (a) 5, (b) 10, and (c) 15 kHz. No notable improvement in precision or resolution were found to accompany an increase in $f_c$. Colors represent 81 (black squares), 130 (red circles), 237 (blue triangles), and 545 kDa (green stars). Results collected using a single SS-nanopore of 7.5nm in diameter and at least 2,000 events were considered for each data point. Concentration of HA varied between 2.5-5 ng/μL for the different MW. Error bars represent measurement standard deviation.*

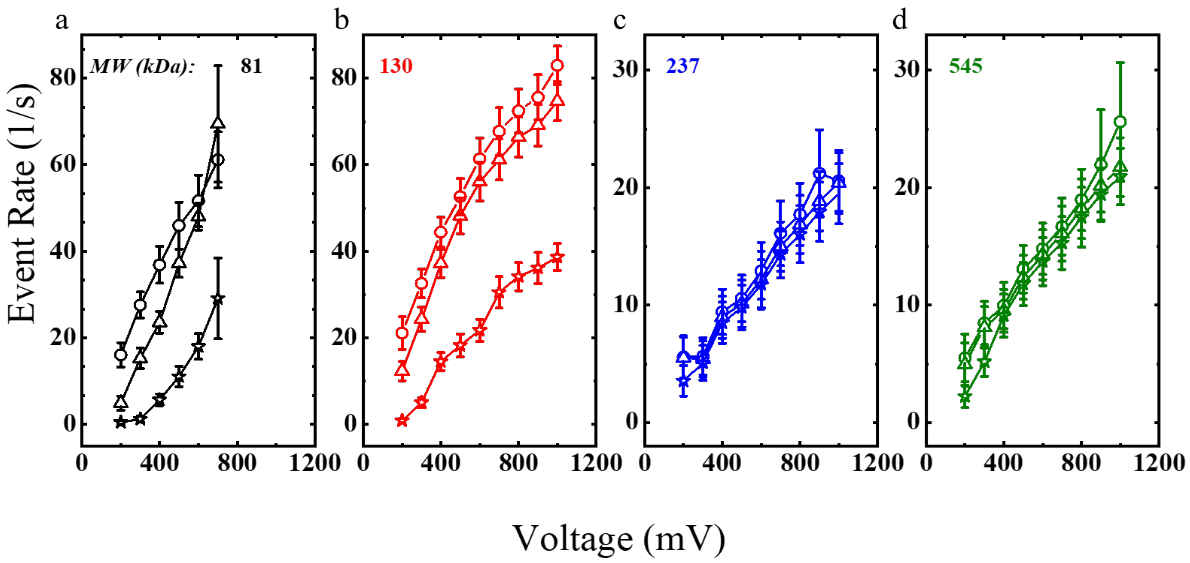


***Supplementary Figure S4.*** *Event rate for quasi-monodisperse HA samples sized (a) 81, (b) 130, (c) 237, and (d) 545 kDa as a function of applied voltage while varying low-pass filter frequencies (fc) utilized during analysis (circle =5 kHz; triangle =10 kHz; star =15 kHz). We observed that event rate was only impacted strongly for low MW HAs (81 and 130 kDa) when subjected to high f. Results collected using a single SS-nanopore of 7.5 nm in diameter and at least 2,000 events were considered for each data point. HA concentration between 2.5-5 ng/µL were used.*

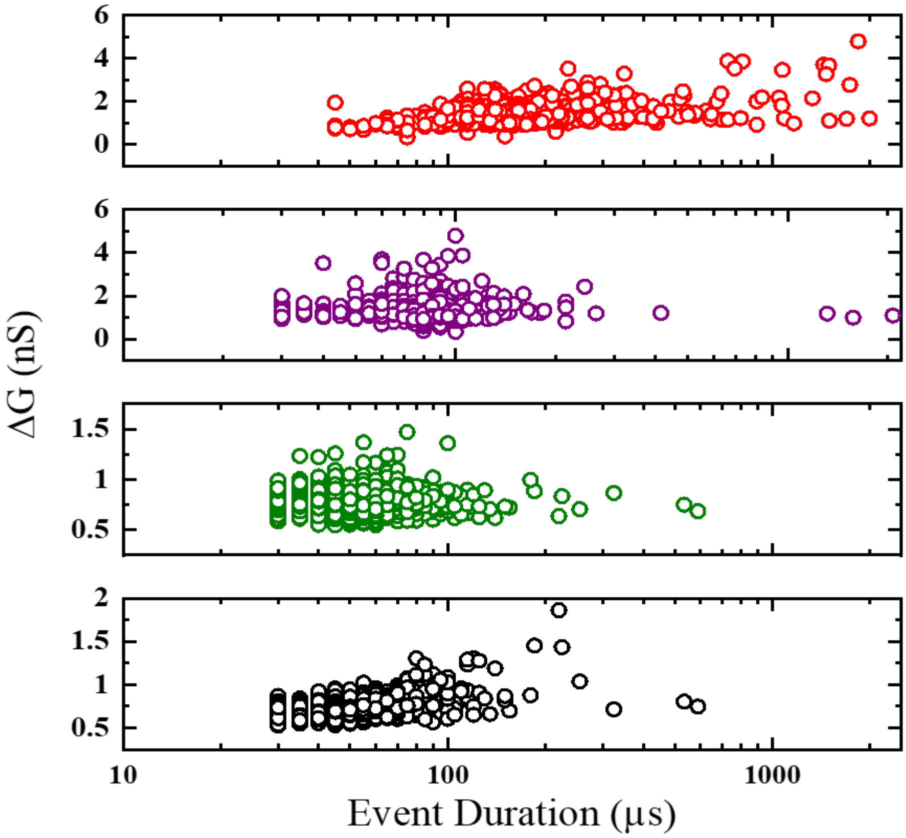


***Supplementary Figure S5***. *(a) Scatter plots of quasi-monodisperse HA of 130 kDa change in conductance with the respective event duration for the recorded individual event properties, when varying the SS-nanopore diameter. (Top to bottom: 3.7, 7.4, 12.9, 19.5 nm) corresponding to the inset to Figure 3 in the main text. The HA concentration was 15 ng/μL and at least 1,500 events were considered for each plot. Data collected at 200 kHz bandwidth with a 100 kHz Bessel filter and analyzed following the application of an additional 5 kHz low-pass filter.*

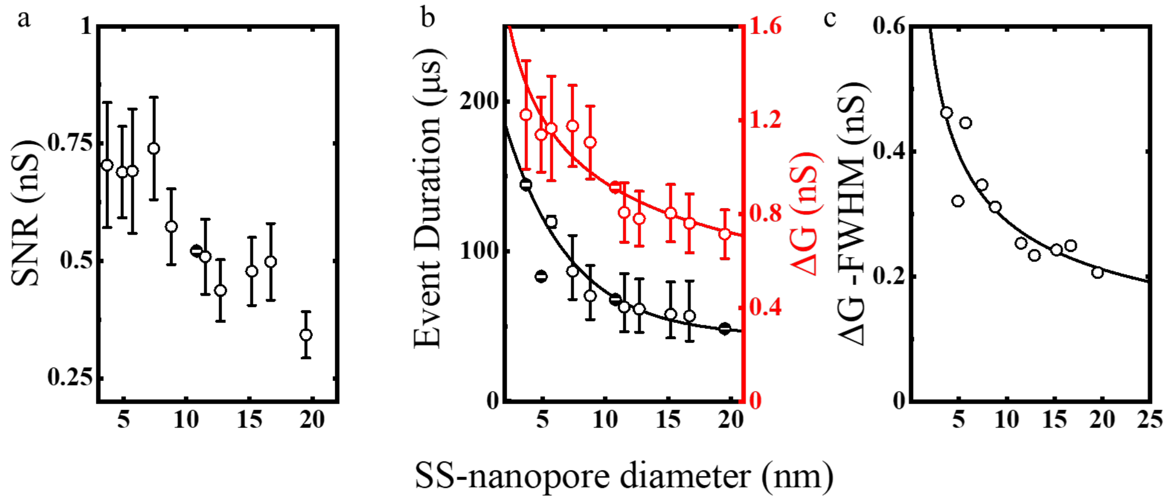


***Supplementary Figure S6.*** *(a) Effect of varying SS-nanopore diameter on signal-to-noise ratio (SNR). The amplitude signal values were derived from log-normal fits to the histogram data of the conductance change for recorded events while noise values were derived from the width of the baseline histogram. Error bars represent standard deviation. (b) Translocation event durations (black) and amplitudes (red) as a function of pore diameter when considered separately, indicating a joint contribution to the decreasing ECD. Error bars represent measurement standard deviation. (c) Full Width Half Max (FWHM) of event amplitude distributions derived from log-normal fits to the data.*

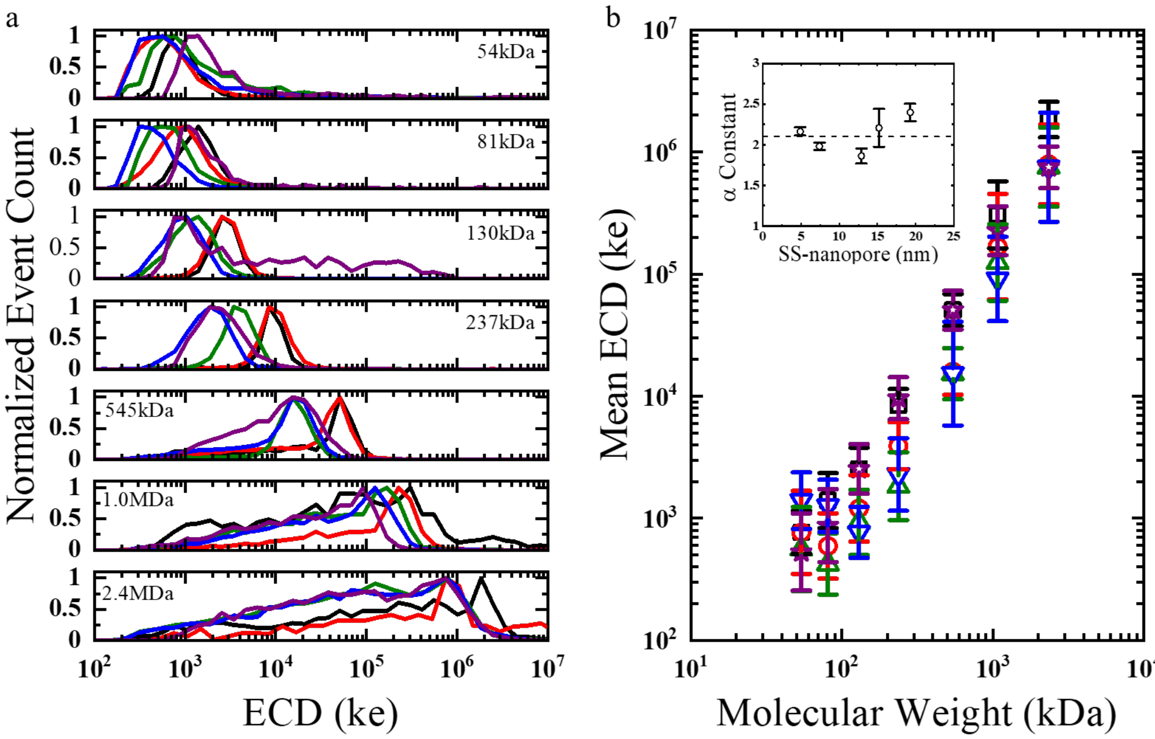


***Supplementary Figure S7.*** *a) ECD distributions for varying quasi-monodisperse HA MWs obtained when utilizing SS-nanopores with diameters of (black/square), 7.4 (red/circle), 12.9 (green/triangle), 15.2 (blue/inverted triangle), 19.3 nm (purple/star). (b) Stacked log-log plot of mean ECD derived from log-normal curve fits to distributions in (a) plotted against HA MW. For samples >1 MDa, a multi-peak fit analysis was performed, and the major peak was used. Inset: The exponent (α) of the power law relationships for all pore diameters studied. α was found to be constant irrespective of pore diameter, yielding a mean value of 2.02 ± 0.16 (dashed line). HA concentrations between 2.5-5.0 ng/μL were used. Error bars represent measurement standard deviation.*

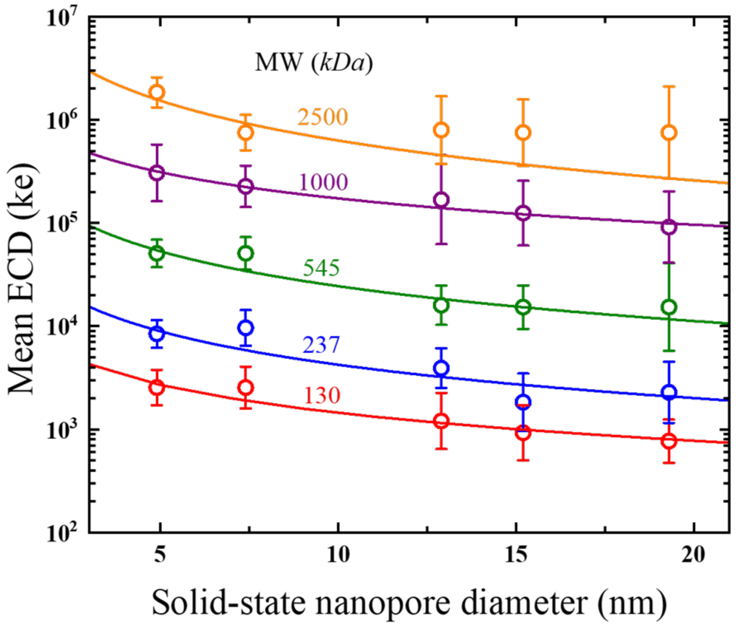


***Supplementary Figure S8.*** *Mean ECD as a function of SS-nanopore diameter for four discrete sizes of quasi-monodisperse HA (Top to bottom: 2500, 1000, 545, 237, or 130 kDa). Solid lines are log-normal fits to the data. All measurements performed in 6 M LiCl buffer at an applied voltage of 200 mV with a HA concentration between 2.5-5 ng/μL. Error bars represent measurement standard deviation.*

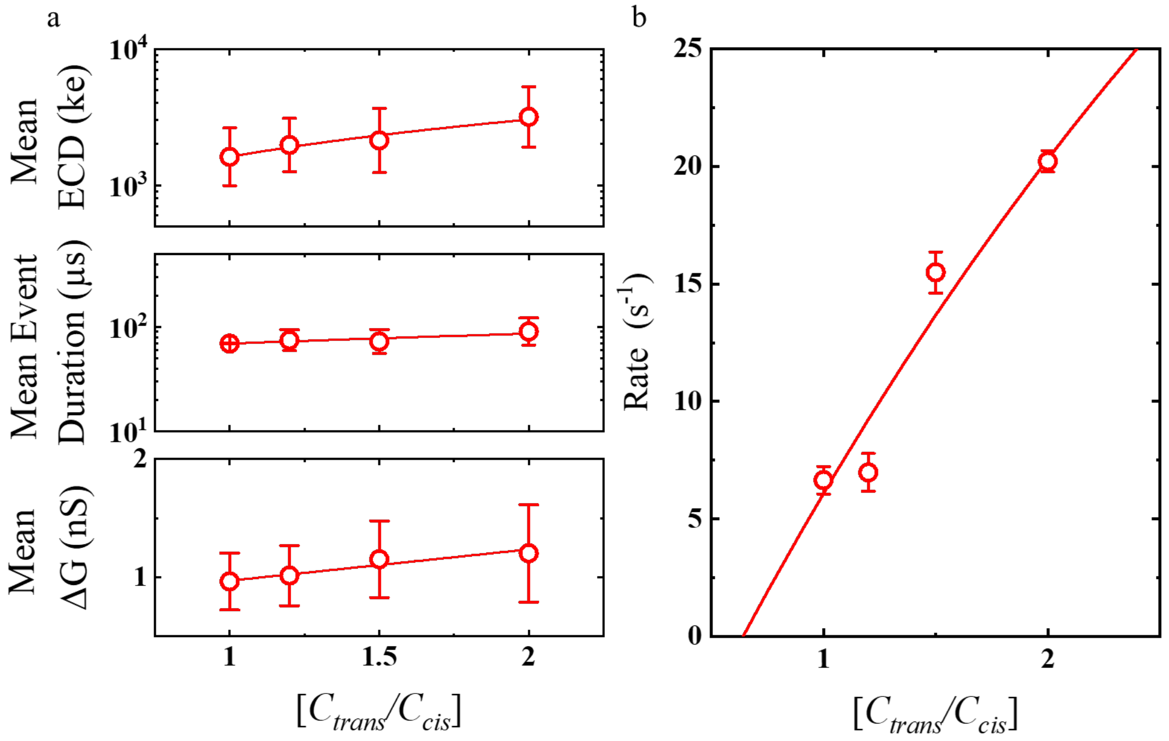


**Supplementary Figure S9.** (*a) Translocation results under varying LiCl asymmetry where $C_{cis}$ is kept at 4 M and $C_{trans}$ is varied from 4 to 8 M, showing mean ECD (top), event duration (middle), and conductance change (bottom). Solid lines are linear fits to the data. (b) Event rates across asymmetric buffer conditions relative to 1:1 [$C_{trans}$:$C_{cis}$] LiCl. Solid line is a logarithmic fit to the data. All measurements were taken at an applied voltage of 200 mV using quasi-monodisperse 237 kDa HA samples prepared at a 2.5 ng/μL concentration***.** *Error bars represent measurement standard deviation.*

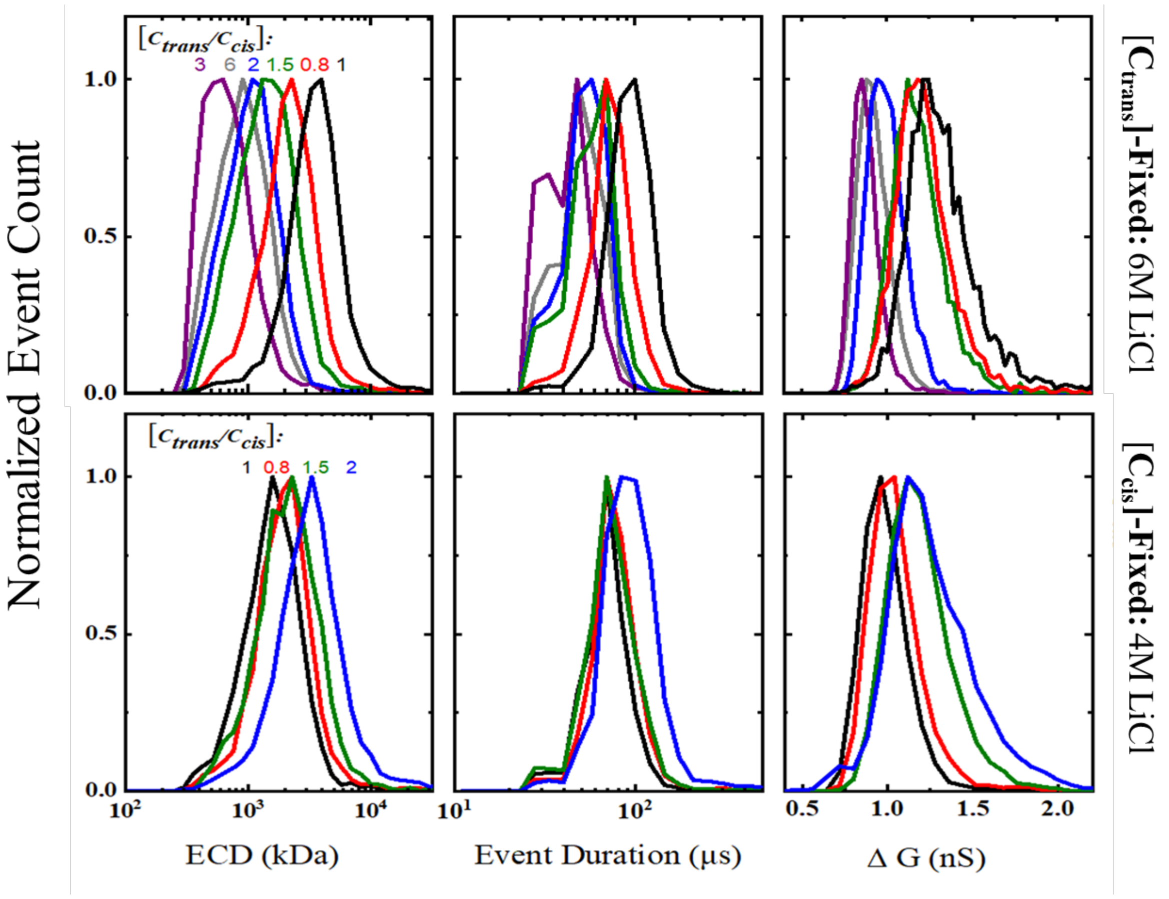


***Supplementary Figure S10.*** *Distributions of ECD (left), dwell time (center), and mean amplitude (right) for 237 kDa quasi-monodisperse HA under varying LiCl salt gradients. Top row shows data from Figure 5b and bottom row shows data from Figure 5c in the main text.*
